# IGS ROTI Maps: Current Status and Its Extension towards Equatorial Region and Southern Hemisphere

**DOI:** 10.3390/s22103748

**Published:** 2022-05-14

**Authors:** Iurii Cherniak, Irina Zakharenkova, Andrzej Krankowski

**Affiliations:** 1 Space Radio-Diagnostic Research Center, University of Warmia and Mazury, 10-719 Olsztyn, Poland; irina.zakharenkova@uwm.edu.pl (I.Z.); kand@uwm.edu.pl (A.K.); 2 COSMIC Program Office, University Corporation for Atmospheric Research, Boulder, CO 80301, USA

**Keywords:** ionosphere, GNSS, ionospheric irregularities, ROTI, geomagnetic storm, auroral oval

## Abstract

The International GNSS Service (IGS) diurnal ROTI maps ionospheric product was developed to characterize ionospheric irregularities occurrence over the Northern hemisphere and has been available for the community since 2014. Currently, the diurnal ROTI maps database hosted by NASA CDDIS covers the period from 2010 to now. Here, we report the ROTI maps product operational status and important changes in the product availability and access. Apart from actual ROTI maps product production, we work on the extension of ROTI maps to cover not only the Northern hemisphere but also the area of the Southern hemisphere and equatorial/low latitude region. Such extended ROTI maps are important for ionospheric irregularities climatology research and ionospheric responses to space weather. We present recent development toward the new ROTI maps product and the updated data format. To evaluate extended the ROTI maps performance, we analyzed the ability to represent key features of ionospheric irregularity occurrence over the Southern hemisphere and low latitudes. For auroral and midlatitudes, we present the cross-comparison of ROTI-derived irregularities patterns over the Northern and Southern hemispheres. For low latitudes, we examined the sensitivity of the resulted ROTI maps to detect plasma irregularities associated with equatorial plasma bubbles development for low, middle, and high solar activity periods.

## 1. Introduction

The Earth’s ionosphere is non-homogeneous plasma that contains fluctuations in the electron density of different magnitudes and spatio-temporal scales. The satellite-to-Earth radio signals can be significantly affected by the disturbed ionosphere. Ionospheric irregularities can lead to strong amplitude and phase scintillations of radio signals used in GPS navigation systems that can affect the operational performance of these systems [1,2,3]. In the high-latitude ionosphere, the development of the intense ionospheric irregularities is primarily controlled by the magnetosphere-ionosphere coupling processes [4]. The high-latitude ionospheric irregularities are generated by different mechanisms, in particular, energetic particle precipitation from the Earth’s magnetosphere [5] and processes of plasma convection [6]. During geomagnetic disturbances, severe ionospheric plasma density irregularities are produced as a result of a significant increase in the auroral particle precipitation, high-latitude ionospheric electric fields, and currents. The storm-induced ionospheric irregularities are responsible for radio wave scintillation and can seriously affect GPS signal propagation. For several decades, ground- and space-based GPS measurements are widely used for the investigation of processes of the ionospheric irregularities’ generation and development within the auroral zone, as well as their impact on the GPS signals during moderate [7] and severe [8,9] geomagnetic storms.

In 2014, the International GNSS Service (IGS) approved a new product “ROTI maps” that allows us to reveal the ionospheric irregularities occurrence at high latitudes using multi-station GPS observations [10]. On a routine basis, the product is now generated by multi-step processing of carrier phase delays in dual-frequency GPS signals and sent to the IGS CDDIS database. This ROTI maps product provides an opportunity to monitor the occurrence and intensity of the high-latitude ionospheric irregularities over the Northern hemisphere. This product is based on raw GPS observations from ~700 representative ground-based GPS stations. The resulted maps show spatial variations of the GPS-based index ROTI (Rate of TEC Index) that are plotted in a polar view projection within a range of 50°–90° N in geomagnetic latitude (MLAT) and 00–24 magnetic local time.

The performance of this ROTI map product for ionosphere plasma irregularities monitoring was demonstrated in representative cases of geomagnetic storms in the 24th solar cycle and validated during the ESA’s (European Space Agency) project MONITOR-2 [11]. The ROTI maps show the occurrence and spatial distribution of high-latitude ionospheric irregularities. The analysis of the ROTI maps during geomagnetic storms of different intensities in 2011–2018 revealed that the ionospheric irregularities formed an oval-like structure that can expand substantially in both the poleward and equatorward directions. The occurrence of the intense storm-induced ionospheric irregularities at middle latitudes can lead to an unexpected degradation of the GPS-based system’s performance.

For further climatological studies of the ionospheric irregularities’ occurrence and spatial distribution and statistical assessment of the Earth’s ionosphere responses to geomagnetic disturbances of different intensities, we propose to extend the actual version of the product towards the equatorial region and the Southern hemisphere.

In recent years, the ground-based segment of GPS permanent stations expanded considerably. It provides a great opportunity to modify and expand the IGS ROTI maps product for a much larger coverage. In this paper, we present a description of the proposed updates and the first results of the updated product for the specification of the ionospheric irregularities occurrence of different origins from high to low latitudes of both hemispheres.

## 2. Data and Methodology

The general methodology for the ROTI maps construction is based on the well-known algorithms of TEC estimation from the frequency-differenced GPS phase delay [12]. During the TEC processing, detection and correction of cycle slips and loss-of-lock were also performed. Monitoring the time-derivative of TEC (ROT, rate of TEC change) is useful for tracing the presence of the ionospheric irregularities. ROT, as a measure of phase fluctuation activity, is calculated using the algorithm, that was proposed by Pi et al. [13]:

(1) $$ROT=\frac{TEC_{k}^{i}-TEC_{k-1}^{i}}{\left(t_{k}-t_{k-1}\right)}$$

where *i* is the visible satellite and *k* is the time of epoch. In standard RINEX files, raw data is sampled every 30 s. ROT is calculated in units of TECU/min for each visible satellite over a GPS station. In order to process RINEX data from GPS receivers, there was used UNAVCO TEQC software for RINEX quality checking and self-developed FORTRAN routines to retrieve the ROT values. The ROT values are calculated and then detrended for all individual satellite tracks with elevation angles over 20 degrees by subtracting the 5-min running average.

The ROTI (Rate of TEC Index) metric can effectively characterize the ionospheric fluctuation activity. ROTI is defined as the standard deviation of the detrended rate of change of TEC (ROT) [13]. Based on the retrieved values of ROT, the ROTI values are calculated as the standard deviation of ROT values over a 5 min interval for each station (Equation (2)):

(2) $$ROTI=\sqrt{\langle ROT^{2}\rangle -{\langle ROT\rangle }^{2}}$$

To observe the spatial behavior of the ionospheric disturbances over the Northern Pole, we visualize the result in the form of an ROTI map. Here, we used a single-layer ionospheric model in which the ionosphere is represented by the thin layer of zero thickness at 350 km height; the locations of the fluctuations are related to the locations of the ionosphere piercing points. Due to strong connections between the Earth’s magnetic field and the ionosphere, the behavior of the fluctuation occurrence is represented as a function of the magnetic local time (MLT) and of the corrected magnetic latitude. Each map, as a daily map, demonstrates ROTI variation with geomagnetic local time (00–24 MLT). ROTI maps are constructed with a grid of 2 deg × 2 deg resolution. The value in every cell is calculated by averaging all ROTI values covered by this cell area and it is proportional to the fluctuation event probability in the current sector. If in the cell there are only a few ROTI values (less than 30 counts), this grid cell is marked as a blank. This cell-by-cell approach allows us to avoid frequent errors in interpolation techniques, related to unrealistic interpolation values over areas with data gaps.

## 3. Results and Discussion

### 3.1. IGS ROTI Maps Product: Current Status

Since 2014, the UWM analysis center for IGS collects and processes data, generates ROTI maps on their basis, and sends them to the IGS server. A software was developed for the regular transfer of observational and navigational files from the IGS, EPN, CORS, and UNAVCO databases. Each day, the raw GPS observations in the RINEX format are downloaded, decompressed, and tested for consistency and quality to fill up the local database. Next, the observational data for a considered day is processed using the ROTI mapping approach as discussed in Section 2. The center provides ROTI maps product that supports regular monitoring of the ionospheric irregularities occurrence over the Northern hemisphere and this product is available for the community via the NASA CDDIS (Crustal Dynamics Data Information System).

Currently, the ROTI maps become available for the period from 2010 to present. Following the IGS Ionosphere Working Group recommendation, the ROTI maps product is accessible at the CDDIS data portal in the same folder “IONEX” such as IONEX TEC GIMs for a particular day. Users can download the ROTI map data file with the name mask rotiDDD0.YYf where the “roti” is a product acronym, DDD stands for the Day-Of-Year (DOY) and YY for the last two-digit of the year. For example, the ROTI map product datafile for the day of 1 January 2022 is marked as roti0010.22f. The reader is referred to [10] for more details on ROTI maps production. The main updates are as follows.

The most important changes happened recently with the NASA CDDIS access rules. Since October 2020, the NASA CDDIS discontinued anonymous ftp access to its archive and moved from simple ftp to secure access protocols (https and ftp-ssl). Now for data assessment, user accounts and new secure access protocols are mandatorily required. The data samples may be downloaded by following the links: https://cddis.nasa.gov/Data_and_Derived_Products/CDDIS_Archive_Access.html (accessed on 27 April 2022) and https://cddis.nasa.gov/About/CDDIS_File_Download_FAQ.html (accessed on 27 April 2022). These changes were also accounted for in the UWM ROTI processing as part of procedures for download of the observational and navigational files from the IGS network hosted at the NASA CDDIS. When operating UWM ROTI maps processing we continuously monitor the availability and data latency of GPS RINEX observations over the Northern hemisphere in IGS, UNAVCO, CORS, and EPN networks. As a result, roughly 700 permanent GPS stations are selected to serve as an input database and we update the list of selected representative stations on a yearly basis to keep a consistent amount and distribution of core observations.

**Figure 1:**
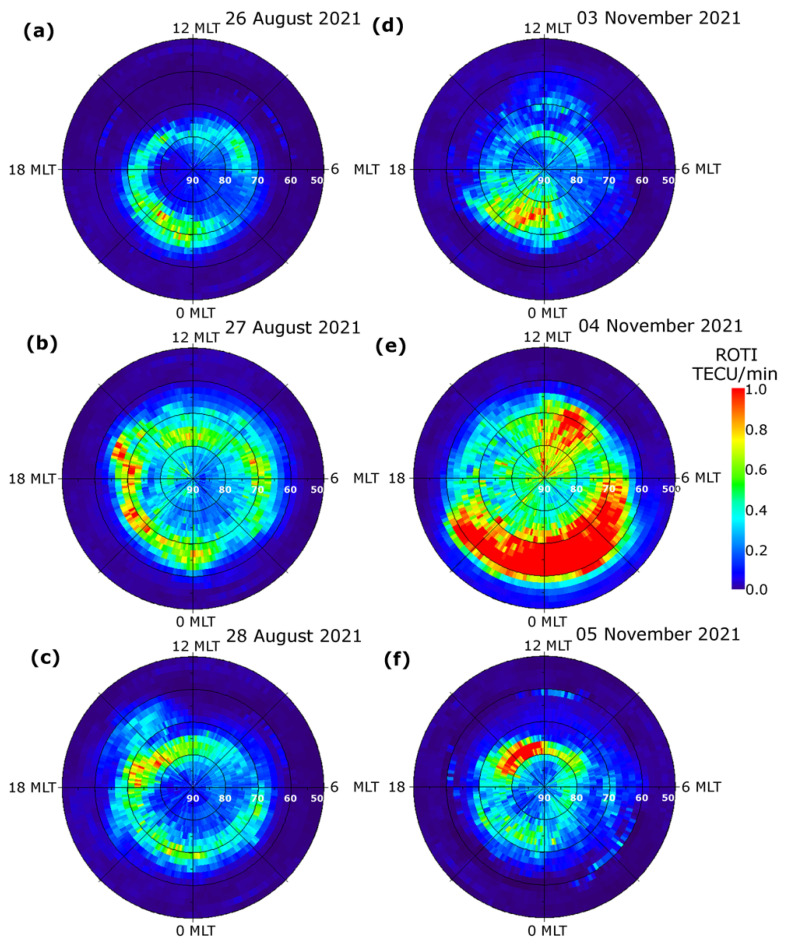
IGS ROTI maps for the Northern hemisphere during two geomagnetic storms that occurred in August 2021 (left panels **a**–**c**) and November 2021 (right panels **d**–**f**). The maps cover 50–90° N MLAT; magnetic local noon/midnight is at the top/bottom, and dusk/dawn is toward the left/right.

To demonstrate the performance of the product under different geomagnetic conditions, we presented a series of the visualized IGS ROTI maps product for the Northern hemisphere during two geomagnetic storms of the 25th solar cycle (Figure 1). The first geomagnetic storm of 27–28 August 2021 was associated with the 26 August C3-class flare and the following coronal mass ejection (CME) arrival. The IMF Bz southward turning occurred after 13 UT on 27 August 2021 and the IMF Bz remained steady negative for more than 14 h till ~04 UT on 28 August 2021. During that time, the main phase of the storm developed starting from ~13 UT on 26 August 2021; the Dst index reached its minimum value of –90 nT at 00:25 UT on 28 August 2021. The highest value of the Kp index for this storm was 5 at 00 UT on 28 August 2021. The auroral activity level increased significantly on 27 August 2021 when the auroral electrojet index AE index reached the values of more than 1000 nT with isolated peaks up to 1500–2000 nT. On the ROTI map corresponding to 26 August 2021 (Figure 1a), one can recognize a belt-like structure with ionospheric irregularities occurrence of relatively weak intensity as revealed by ROTI near 70–78° N MLAT. We should note that these pre-storm conditions were weakly disturbed due to the previous geomagnetic disturbance that occurred on 25 August 2021. Under such conditions, the ionospheric irregularities detected by ROTI at high latitudes can be produced by auroral particle precipitations from the disturbed magnetosphere. On the next day, 27 August 2021 (Figure 1b), when the main phase of the storm was developed the ROTI map allowed us to detect the intensification of the ionospheric irregularities development and extension of irregularity oval from ~70–75° MLAT towards lower latitudes at ~65° MLAT in both the dayside and nightside sectors. In the afternoon and evening sectors, the intensity of irregularities exceeded 0.6 TECU/min. The ROTI map constructed for the recovery phase on 28 August 2021 (Figure 1c), shows the presence of the ionospheric irregularities with a moderate intensity level of ~0.5 TECU/min, and the irregularities oval shrank with its equatorward border located at ~70° MLAT at the night sector but it did not reach the pre-storm level at the dayside sector.

The geomagnetic storm of 4 November 2021 was classified as a G3—strong geomagnetic storm. As it was reported by the National Oceanic and Atmospheric Administration (NOAA) Space Weather Prediction Center, this storm was related CMEs that occurred on 1–2 November 2021 and arrived with the high-speed solar wind in the Earth magnetosphere on 3 November 2021. During the main phase of the storm on 4 November, the Kp index reached the maximal value of 8- and the Dst index dropped down to −118 nT. Aurora lights produced by this storm were reported for many locations in USA and Canada in the Northern hemisphere and in New Zealand in the Southern one. Figure 1d–f presents a series of the daily ROTI maps illustrating the occurrence and intensity of the high-latitude ionospheric irregularities for the period of 3–5 November 2021. These ROTI maps for the Northern hemisphere illustrate an evolution of the ionospheric fluctuation pattern before the storm’s commencement (3 November, panel d), large intensification of ionospheric irregularities during the main phase of the storm (4 November, panel e), and ionospheric conditions at the recovery phase of geomagnetic disturbance (5 November, panel f). On the day before the storm, the intense irregularities were located mainly in the nighttime sector (20–00 MLT) within the latitudinal range of 70–80° MLAT, and their occurrence can be related to the quiet-time particle precipitation from the magnetotail into the nightside ionosphere. During the main phase of the storm, it was registered a strong intensification of plasma irregularities characterized by the ROTI values increasing up to 1.0 TECU/min (which indicates the presence of strong ionospheric plasma gradients), and the equatorward border of the irregularities oval expanded towards 55° MLAT in the nightside sector. On the ROTI map constructed for 4 November 2021, besides the large expansion of the irregularities oval one can note the presence of the structures elongated from dayside (9–12 MLT sector) towards the geomagnetic pole that can be associated with the formation of the SED–TOI (storm-enhanced density and tongue of ionization) structures which follow the convection pattern anti-sunward across the polar cap region. During the recovery phase of the geomagnetic storm, the fluctuation activity pattern on 5 November 2021 (panel f) revealed a considerable poleward shrinking of the zone with intense ionospheric irregularities, and they were registered again mainly near the cusp area and in the evening sector.

### 3.2. IGS ROTI Maps Product: Database Extension

It is well-known that ionospheric plasma density irregularities that occurred at auroral latitudes and equatorial regions under quiet and disturbed conditions have quite different morphology and different physical mechanisms behind them. For the sake of further climatological studies of the ionospheric irregularities’ occurrence and spatial distribution and statistical assessment of the Earth’s ionosphere responses to geomagnetic conditions of different intensities, we propose to extend the actual version of the product towards the equatorial region and the Southern hemisphere. In recent years, the ground-based segment of GPS permanent stations expanded considerably. It provides a great opportunity to modify and expand the IGS ROTI maps product for a much larger coverage of the equatorial region and the Southern hemisphere.

**Figure 2:**
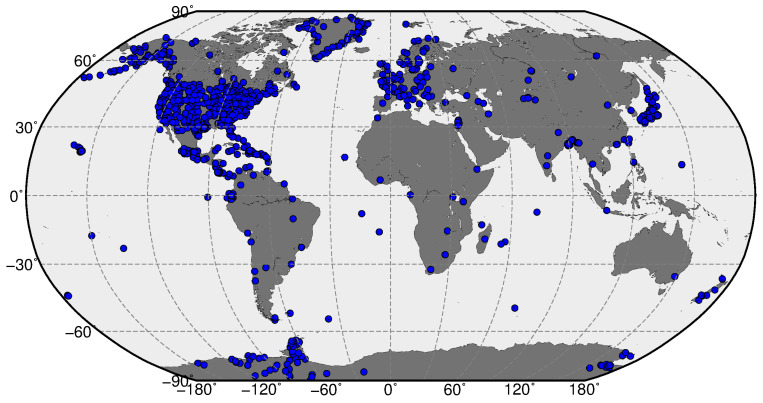
Geographical location of the GPS stations (blue dots) from UNAVCO, IGS, CORS, SONEL, and EPN networks selected as the dataset for the construction of the extended ROTI maps.

Figure 2 illustrates a geographical distribution of ~1200 reference ground-based GPS stations operated by IGS, UNAVCO, CORS, EPN, and SOPAC networks and provided GPS observations (daily observational and navigational files) with low latency (24–48 h). The observational data from these stations in the standard RINEX 2.0 format are available for the users.

We should note that the ground-based GPS stations are distributed non-uniformly around the globe. In particular, the major part of GPS stations operate at midlatitudes of the Northern hemisphere. In the equatorial region, there are available GPS observations from ~400 stations, and for the Southern hemisphere’s middle and high latitudes we have only ~150 GPS stations that provided continuous and low latency observations. One of the important steps toward this goal is to modify data acquisition and data processing procedures in order to get ROTI maps with proper coverage of the new regions (the Southern hemisphere and equatorial region) and performance similar to the actual ROTI product for the Northern hemisphere. In particular, we included in the core dataset for ROTI calculation the GPS observations freely available from the CORS and SONEL networks that cover numerous areas over the equatorial region. Additionally, for these areas with fewer GPS observations, we decrease the threshold number of minimal accumulated data points from a grid cell from 30 to 10. For areas of the ROTI maps extension, we mark a grid cell as “no data” when the number of observation data points is less than 10.

### 3.3. Extension of the IGS ROTI Maps Product: Performance Evaluation

**Figure 3:**
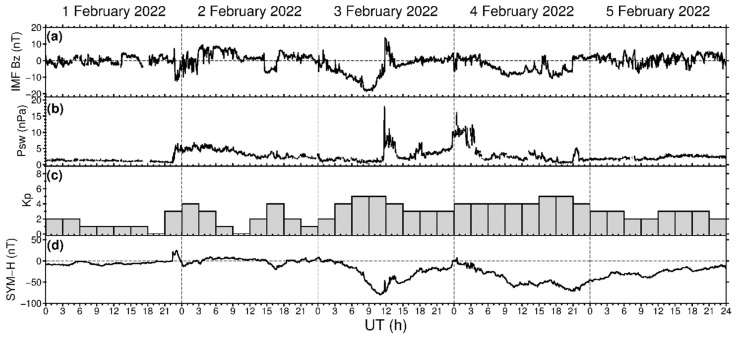
Geomagnetic conditions during 1–5 February 2022: (**a**) IMF Bz component, (**b**) dynamic pressure of the solar wind, (**c**) Kp index, and (**d**) SYM-H index.

**Figure 4:**
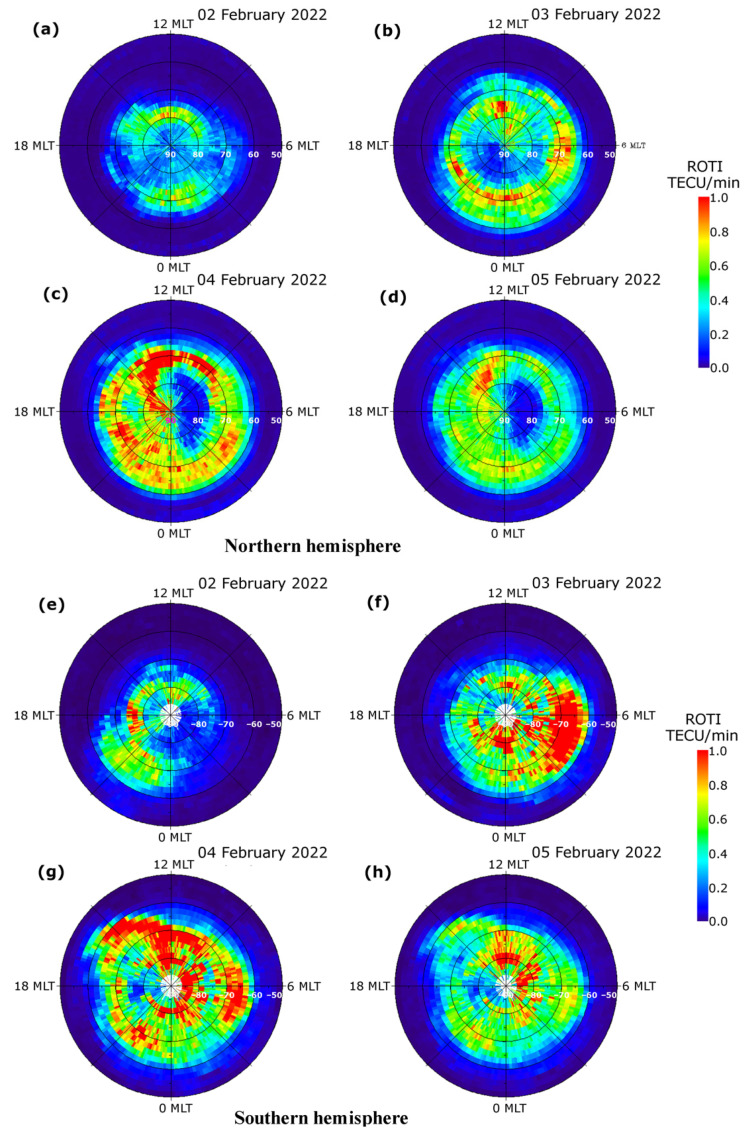
Daily GPS ROTI maps constructed for the Northern (**a**–**d**) and Southern (**e**–**h**) hemispheres for the period between 2 February and 5 February 2022. The maps cover 50°–90° N MLAT for Northern and 50°–90° S MLAT for Southern hemispheres.

The performance of the new ROTI maps for the Southern hemisphere and equatorial region can be assessed by their ability to represent the well-known features of ionospheric irregularities development over these areas. To demonstrate the performance of the ROTI maps over high/middle latitudes of the Southern hemisphere, we carried out the comparative study of irregularities development specified by ROTI maps for both the Northern and Southern hemispheres for the case of the recent geomagnetic storm that occurred in February 2022. Figure 3 presents temporal changes in the major geophysical parameters during 1–5 February 2022. The resultant product was visualized in the form of a series of ROTI maps in polar projections for both the Northern and Southern hemispheres (Figure 4). The visualization grid size is 2° MLAT by 0.13 h (8 min) MLT, which corresponds to 20 × 180 sector bins. We utilized a color scale ranging from 0.0 to 1.0 TECU/min, where the ROTI values below 0.2 TECU/min, specified by dark blue color, represent very weak or absence of ionospheric irregularities, whereas the ROTI values higher than 0.8–1.0 TECU/min, specified by orange and red color, indicate the presence of strong ionospheric irregularities within that particular cell. In maps of the Southern hemisphere, we have “no data” area marked by white color due to an absence or insufficient observational data over the Southern magnetic pole.

The first CME arrived at ~22:20 UT on 1 February 2022 which caused geomagnetic field disturbances with a rapid increase of the Dst/SYM-H index from 0 to 25 nT and an increase of the Kp index to 3–4 (Figure 3). Intensification of geomagnetic activity led to an occurrence of high-latitude ionospheric irregularities due to auroral particle precipitation and high-latitude ionospheric electric fields. For 2 February 2022, the ionospheric irregularities were clearly detected at high latitudes; they were localized mainly at ~75–80° N MLAT in the dayside and at ~70° MLAT in the nightside sector of the Northern hemisphere (Figure 4a). Over the Southern hemisphere (Figure 4e), localization of the ionospheric irregularities in the dayside sector was quite similar to the Northern hemisphere, but in the nightside sector, the zone of noticeable irregularities was shifted from midnight to 20–21 MLT. On 3 February 2022, the main phase of the geomagnetic storm (identified by the SYM-H index) developed with the SYM-H index decrease reaching its minimal value of –80 nT at ~11 UT, whereas the Kp index reached 5 (Figure 3). The series of the next maps corresponded to a period of increased geomagnetic activity. In comparison with observations for the previous day, the daily ROTI maps for 3 February 2022 demonstrate a significant intensification of ionospheric irregularities occurrence with ROTI values exceeding 0.9–1.0 TECU/min over both hemispheres, as well as a simultaneous expansion of the irregularities oval area in the poleward and equatorward directions (Figure 4b,f).

The irregularities oval expanded equatorward down to 60° N MLAT in the nightside sector, and the location of its equatorward edge is in the vicinity of the main ionospheric trough and the auroral oval equatorial edge. In the dayside sector (top part of the polar maps), the strong ionospheric irregularities were detected over 75°–80° N MLAT, which can be related to the dayside aurora and particle precipitation into the dayside cusp. The poleward-oriented structures in both the dayside and nightside sectors can represent signatures of the storm-induced ionospheric plasma dynamic processes, such as the polar cap patches that follow the convection pattern across the polar cap. The pronounced hemispheric asymmetry is registered in the morning sector (03–08 MLT) with a more intense irregularity occurrence in the Southern hemisphere. On 4 February 2022, the second geomagnetic storm developed after a short recovery phase of the first storm.

**Figure 5:**
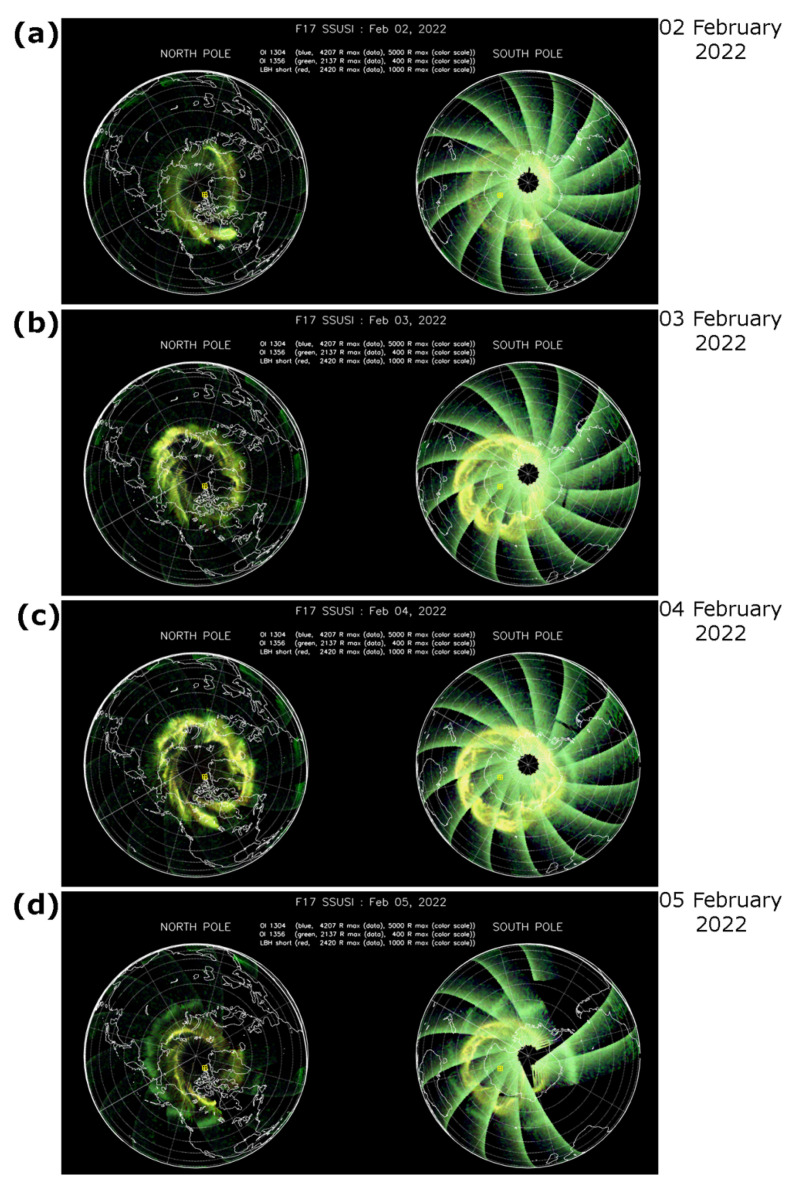
Daily summary auroral images from the Special Sensor Ultraviolet Scanning Imager (SSUSI) sensor onboard DMSP F17 satellite with a polar map projection for (**a**–**d**) 2–5 February 2021 (https://ssusi.jhuapl.edu/images_daily_l1b, accessed on 27 April 2022).

Additionally, we examined the optical observation of the ionosphere by the Special Sensor Ultraviolet Spectrographic Imager (SSUSI) which measures far ultraviolet emissions in five different wavelength bands (HI 121.6 nm, OI 130.4 nm, OI 135.6 nm, N2 LBHS (140–150 nm) and N2 LBHL (165–180 nm)) from the Earth’s upper atmosphere [14]. These channels capture the main auroral UV emissions. Figure 5 shows summary SSUSI images as a daily accumulation of all nightside passes of the DMSP F17 satellite across the Northern and Southern Pole regions during 2–5 February 2022. For 2 February (Figure 5a), the SSUSI-detected auroral emissions were localized at high latitudes with the brightest emission over Canada in the Northern hemisphere and near the Antarctica continent in the Southern one. On 3 February (Figure 5b), when the first geomagnetic storm developed, the overall brightness of auroral emission increased in comparison with the previous, comparatively quiet day, and the oval-like shape of the aurora was clearly seen expanded in size even at the background of the sunlight summer conditions over Antarctica in the Southern hemisphere. One can see an expansion of auroral oval towards lower latitudes: for the Northern hemisphere—towards the south of Greenland, Iceland, north of Russia, for the Southern hemisphere—mostly towards Australia and New Zealand. For 4 February (Figure 5c) the aurora brightness was also high and the auroral oval expanded in the similar directions as on the previous day. For 5 February (panel c), the brightness of auroral emissions captured by SUSSI was decreased and the auroral oval size returned practically to the pre-storm conditions of 2 February 2022. So, aurora evolution tracked by the SUSSI instrument has a good consistency with ionospheric irregularities oval behavior represented by ROTI maps—rather weak and narrow on 2 February 2022, intensification during two geomagnetic storms developed on 3 and 4 February 2022, and return to the more quiet conditions on 5 February 2022.

The 3–4 February 2022 event was a moderate geomagnetic storm, but this event had a significant impact on the about 40 SpaceX satellites that were launched on 3 February 2022, and the next day they reentered the Earth’s atmosphere, as was reported by SpaceX. One of the possible reasons can be related to an increased satellite drag due to thermospheric heating. The effects of thermosphere density increased due to thermosphere heating and impact on satellite drag is a well-known phenomenon reported in [15,16,17,18]. During geomagnetic storms, the predominant sources of this thermosphere heating are Joule heating by electrical currents and particle precipitation from the magnetosphere [19]. The initial response at high latitude is that Joule heating raises the temperature of the upper thermosphere and ion drag drives high-velocity neutral winds. The energy comes to the thermosphere primarily through high latitudes and propagates towards the equatorial region with gravity waves and wind surges [20]. Large-scale traveling ionospheric disturbances (LSTIDs) represent an ionospheric manifestation of this phenomenon. In [21], with joint analysis of the ionospheric plasma irregularities, FACs, and LSTIDs reveals that a zone with the intense FACs and auroral ionospheric irregularities we reported that the area where this thermosphere heating can occur (LSTIDs excitation zone) is in the same time and location where most intensive ionospheric plasma irregularities detected by the ROTI technique are developed. The plasma irregularities produced by particles precipitation and magnetospheric fields and currents which parts of the energy deposition process. The ROTI-specified irregularities oval and extension of this oval towards midlatitudes are related with the start of magnetospheric energy deposited to the thermosphere intensification with following atmospheric waves propagation towards the equator, increased thermosphere density, and corresponded satellite drag. So, the development of intense ionospheric irregularities at high/suboral altitudes indicated the first chain of processes leading to satellite drag increase, and the ROTI maps product could help to reconstruct the conditions leading to drag enhancement in a space-weather-oriented framework.

The polar ROTI maps corresponded to the most active periods of these geomagnetic storms for 3 and 4 February 2022 (Figure 4) clearly demonstrate the occurrence of intense ionospheric irregularities in the form of a large-scale oval extended as far equatorward as 60° MLAT in both hemispheres. The high-latitude ionospheric irregularities were driven by an enhanced auroral activity due to auroral particles’ precipitation, ionospheric conductivity, and current enhancements. Presence of signatures of intense ionospheric irregularities and irregularities of oval expansion on the ROTI maps of both hemispheres can be considered not only as threatening conditions for transionospheric radio waves propagation and for GPS performance degradation but also as a proxy for a storm-time atmosphere heating phenomena responsible for atmospheric drag enhancement.

The equatorial ionosphere response to geomagnetic storms is also of interest. During geomagnetic disturbances, the effects of prompt penetration electric fields (PPEFs) and disturbance dynamo electric fields (DDEFs) can largely transform the occurrence of typical equatorial plasma bubbles. Generally attributed to the sudden southward turn of the IMF Bz, the PPEFs appeared in the equatorial region instantaneously [22,23]. The PPEFs are usually eastward during daytime (upward drift), and its superposition with the normal pre-reversal enhancement can produce much larger upward vertical drifts in the local postsunset sector [24]. The DDEFs need more time to develop (several hours after a storm commencement) and they are long-lived. The DDEFs are driven via global scale thermospheric wind circulation due to Joule heating at high latitudes [25]. Typically, the DDEFs are westward during daytime (downward drift) and eastward at nighttime (upward drift). Therefore, the eastward PPEFs that appeared in the local sunset zone can result in a strong upward plasma drift that led to storm-induced equatorial plasma bubbles development. With further storm development, the dominating DDEFs can cause downward plasma drift in the local sunset regions that result in the suppression of postsunset equatorial plasma bubbles during the storm’s recovery phase.

**Figure 6:**
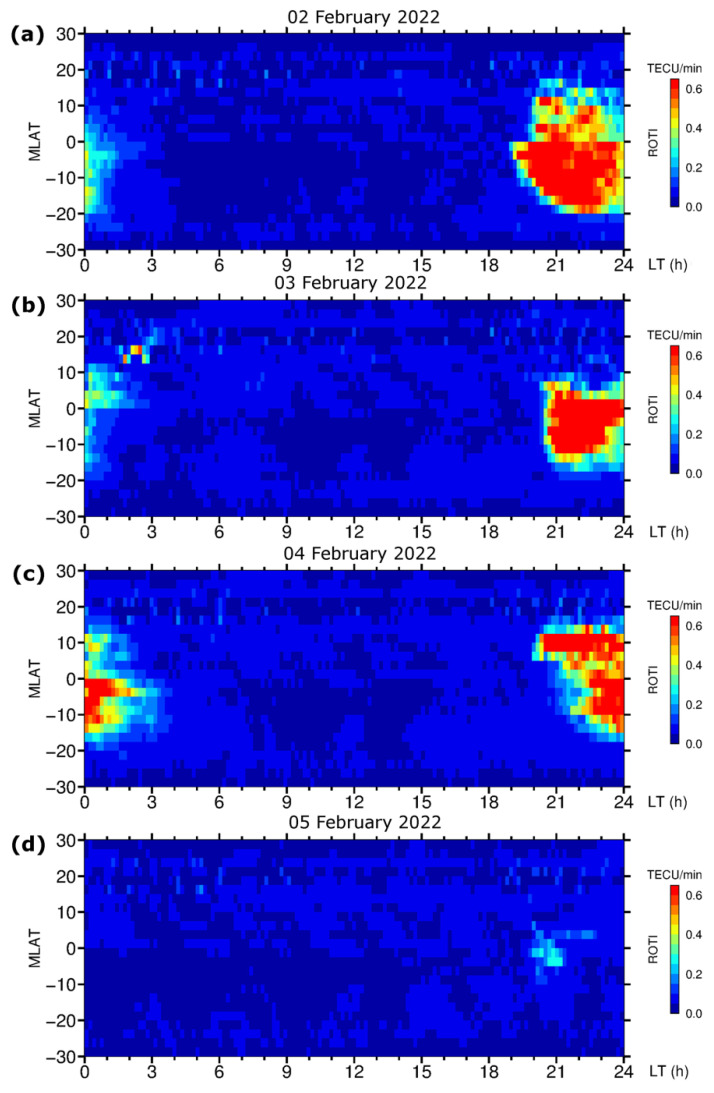
Daily GPS ROTI maps constructed for the equatorial region for (**a**–**d**) 2–5 February 2022. Maps are plotted in MLAT vs. MLT coordinates and cover 30° S–30° N MLAT and 00–24 MLT.

Figure 6 presents a day-by-day sequence of the ROTI maps for the equatorial region for 2–5 February 2022. The maps indicated the occurrence of intense equatorial ionospheric irregularities in the local postsunset period after ~19 LT. Their latitudinal extension was within ±20° MLAT. For 2 February 2022, the equatorial irregularities dissipated close to the local midnight (00–01 LT). For the first geomagnetic storm on 3 February 2022, the signatures of the ionospheric irregularities were detected further in the nighttime till 02–03 LT. For 4 February 2022, during the next phase of the geomagnetic storm, the ROTI map shows the presence of ionospheric irregularities mainly after 21LT (2 h later than on the less disturbed day of 2 February) and they persisted for a much longer period after midnight, up to 3 LT and had a much higher intensity comparing to the previous days. For 5 February 2022, during the recovery phase, we can see strong suppression of the postsunset equatorial ionospheric irregularities most possibly associated with the action of the storm-induced DDEFs. This variability was driven by complex processes that affected the equatorial ionosphere during a geomagnetic storm.

**Figure 7:**
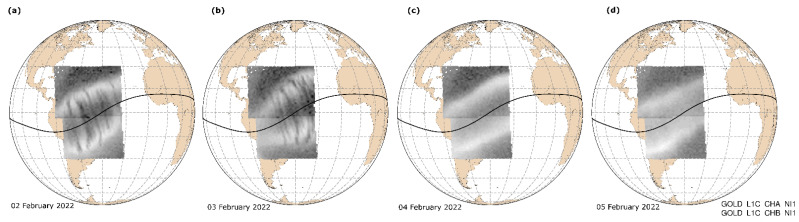
Examples of the GOLD images for (**a–d**) 2–5 February 2022. Dark narrow vertical strips represent signatures of the equatorial plasma bubbles that occurred in the South American sector.

The ionospheric plasma depletion associated with the equatorial plasma bubbles development can be detected using NASA’s Global-scale Observations of the Limb and Disk (GOLD) instrument from the geostationary orbit by analyzing Earth’s airglow emissions [26]. The series of GOLD images in Figure 7 demonstrates examples of the GOLD detection of the equatorial plasma bubbles over the South America sector during the considered period in February 2022. The equatorial plasma bubbles signatures (dark narrow C-shape strips seen at both sides from the magnetic equator marked by a solid black line) were well revealed on the days 2 and 3 February 2022. During the next days, 3 and 4 February 2022, the GOLD images show suppression of the equatorial plasma bubbles during that time. These independent observations are in good agreement with the effects captured by ROTI maps, where we also can see intense irregularities at postsunset hours on 2 and 3 February 2022, and graduate decrease of the irregularities’ occurrence on 4 February 2022, and suppression on 5 February 2022. The presence of irregularities on the ROTI map corresponded to 4 February, which is different from the GOLD results, can be explained by the fact that the daily ROTI map was constructed using observations from GPS stations at all longitudes, whereas GOLD was focused on the American-Atlantic sector.

For further analysis of the extended ROTI maps performance constructed for the equatorial region, we focused on the periods that corresponded to the different conditions for equatorial irregularities development. From the earlier studies on the climatology of equatorial plasma bubbles development responsible for the occurrence of intense ionospheric irregularities in the equatorial region, there are determined so-called “bubble seasons” that can be different for different longitudinal sectors. Under the quiet conditions, high bubble season can be normally attributed to periods close to the fall/spring equinoxes and low bubble season corresponds to the June solstice practically for all sectors. The important feature of the equatorial plasma bubbles occurrence is their seeding and development during local postsunset hours and the rate of the bubbles dissipation after local midnight. However, during geomagnetic disturbances prompt penetration of the magnetospheric electrical fields can create favorable conditions for the plasma bubbles development even during a low bubble season, as well as outside postsunset hours, e.g., after midnight and pre-sunrise hours.

**Figure 8:**
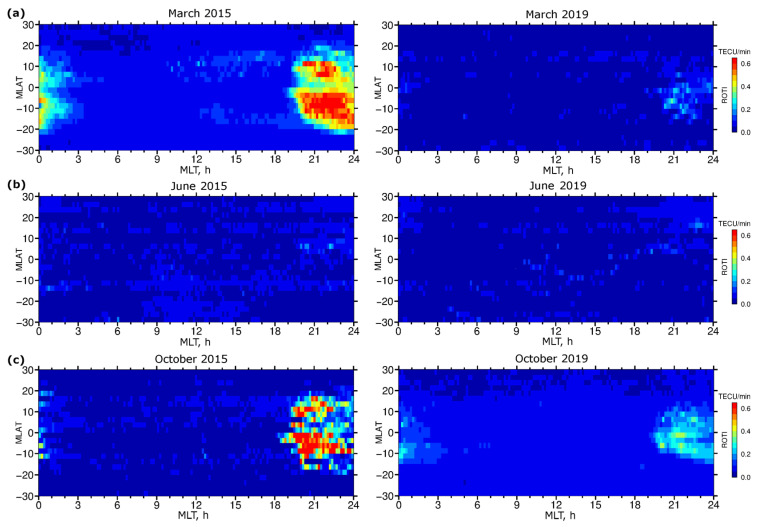
Daily GPS ROTI maps constructed for the equatorial region for (**a**) March, (**b**) June, and (**c**) October at high (2015, **left panels**) and low (2019, **right panels**) levels of solar activity.

Figure 8 presents diurnal ROTI maps constructed for the equatorial region and low latitudes (30° S–30° N MLAT) for the representative periods of March, June, and October at high (2015) and low (2019) levels of solar activity. For the examined periods, the ROTI maps allow us to recognize the occurrence of the ionospheric irregularities associated with the equatorial plasma bubble phenomenon during local postsunset hours, i.e., after ~19 LT. The pre-reversal enhancement of the postsunset vertical plasma drift led to the growth of the Rayleigh–Taylor instability resulting in equatorial plasma bubbles development. Typically, the equatorial plasma bubbles are generated after sunset and the peak height of the bubble rise above the magnetic equator (apex altitude) can determine the latitudinal extent of the bubble from the magnetic equator. The ROTI maps revealed the presence of ionospheric irregularities as detected by GPS ROTI on both sides of the magnetic equator. The ROTI maps also show some differences in the irregularities’ patterns for the considered period, in particular intensity, time of irregularities onset, and their persistence after local midnight. In these examples, the most intense and prolonged in time ionospheric irregularities developed during the high “bubble season” in the solar maximum period (Figure 8a,c, left panels), and the weaker signatures were registered during the low solar activity period (Figure 8a,c, right panels). June solstice is traditionally considered a low “bubble season” period with the lowest probability to observe postsunset equatorial plasma bubbles around the globe e.g., [27,28]. Figure 8b illustrates well the lowest intensity of the ROTI values in the equatorial region during both the high (June 2015) and low (June 2019) solar activity. The intensity, as well as latitudinal and temporal extensions of equatorial ionospheric irregularities detected by ROTI maps, are related to the increase of background electron density due to solar flux variations. The ROTI maps are sensitive to reproducing such types of ionospheric irregularities variations.

### 3.4. Extension of the IGS ROTI Maps Product towards the Equatorial Region and the Southern Hemisphere: A New Format

During the IGS ROTI maps product preparation phase, the format for the ROTI maps product was agreed [29]. This format is based on the standard IONEX format of the well-known ionospheric product GIM (Global Ionospheric Map). The name of the product file is set as “rotiDDD0.YYf” which corresponds to the product type acronym “roti”, DDD stands for the Day-Of-Year (DOY), and the file extension YY—the last two-digit for the year. For the extended ROTI maps product, we propose to leave the name structure the same, only indicate “rotiexDDD0.YYf” as the extended product. We also need to change the internal structure of the ROTI map file both in the header and data section regarding the IGS ROTI maps for the Northern hemisphere only. The header should include the updated information about the type of the product such as the ROTI map for the Northern, Southern hemisphere, and equatorial areas. The name of the processing center—UWM—does not change. We updated basic information on the database and put a new description of the map’s grid for the Northern, and Southern hemispheres, and equatorial parts.

**Figure 9:**
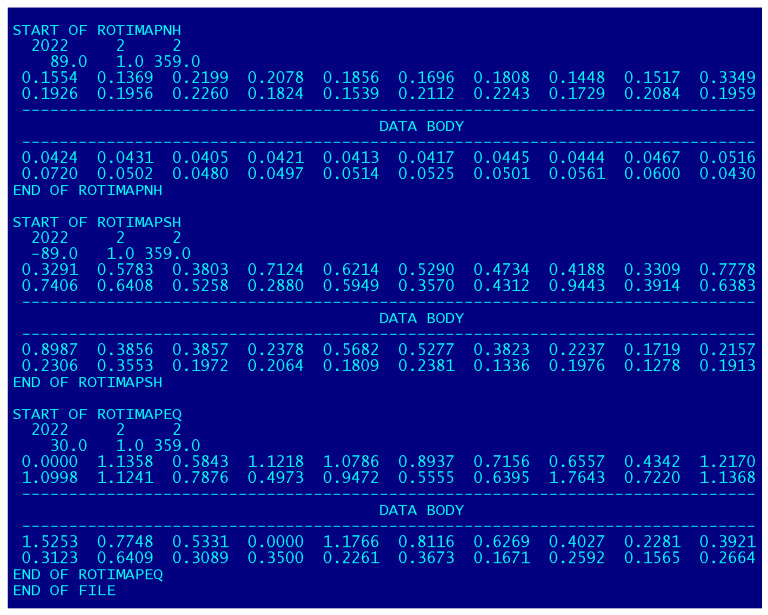
Format of the ASCII file with the extended version of the IGS ROTI map product.

Similar to the main our product, official IGS ROTI maps for the Northern hemisphere, in the new extended ROTI maps version the data body part is indicated by the keyword “START OF ROTIMAP NH” for the Northern hemisphere section, and then it follows by information about the date of the particular ROTI map (Figure 9). Then, it follows by the data block with the ROTI values in an MLAT vs. MLT grid for this day, and the ROTI values are presented in TECU/min units. This grid is the same equidistant grid such as one in the main IGS ROTI maps for the Northern hemisphere product and covers from 00 MLT to 24 MLT and from 50° MLAT to 90° MLAT with an increment of 2° MLAT and of 8 min in time. The ROTI value for every bin is centered on the corresponding grid node. This section is closed by keyword “STOP OF ROTIMAP NH”. We should note that this Northern hemisphere section body’s format does not change significantly and users interested in irregularity specifications over the Northern hemisphere can work with the extended product similar to the main version of the IGS ROTI maps.

The next section corresponds to the Southern hemisphere section marked by the keyword “START OF ROTIPMAP SH” with information about the date of this particular Southern hemisphere ROTI polar map. Following the data block for the Southern hemisphere presented again with MLAT vs. MLT grided ROTI values for this day. The structure is the same as for the Northern hemisphere section and it covers from 00 MLT to 24 MLT with an increment of 2° MLAT and of 8 min in time but the only difference is that this gridded data covers Southern hemisphere high and middle latitudes from −50° MLAT to −90° MLAT. This section is also closed by the keyword “STOP OF ROTIMAP SH”.

The last section of the extended ROTI maps contains information on irregularity distributions on a daily basis as specified by ROTI for the equatorial region. It is indicated by the keyword “START OF ROTIPMAP EQ”, and contains information about the year and day of the year for this equatorial area ROTI map.

## 4. Conclusions

The IGS diurnal ROTI maps ionospheric product was developed to characterize ionospheric irregularities occurrence over the Northern hemisphere, and it provides a reliable way to specify the storm-induced ionospheric plasma density irregularities large-scale patterns. This ionospheric product has been available for the community since 2014 and now the diurnal ROTI maps database hosted by NASA CDDIS covers the time period from 2010 to the present day. The twelve-year dataset of this product may be used for retrospective analysis of ionospheric responses to space weather disturbances, ionospheric irregularities impact on GNSS positioning and can contribute to the future developments of the ionospheric irregularities occurrence forecasting approaches, as well as serve as a database for the assessment of the ionospheric irregularities models’ performance. In this paper, we provided information about the actual IGS ROTI maps product status and highlight the most important changes in product availability and data access.

Using the ROTI maps for several recent geomagnetic storm cases, we demonstrated how during the space weather events of different intensities of the ionospheric irregularities oval expanded largely in size towards midlatitudes with a simultaneous increase of the irregularities intensity. These effects are associated with an increase in storm-time auroral activity caused by auroral particle precipitation and plasma instabilities generation.

Besides the continuous support of the actual ROTI maps product generation, the UWM team is working on the tasks of extension of ROTI maps to cover not only the Northern hemisphere high and middle latitudes but also the similar area of the Southern hemisphere, as well as equatorial and low latitude region. The ROTI maps with the extended coverage are important for further climatological studies of the ionospheric irregularities’ occurrence and spatial distribution and statistical assessment of the Earth’s ionosphere responses to geomagnetic conditions of different intensities. In recent years, the ground-based segment of GPS permanent stations expanded considerably. It provides a great opportunity to extend the current IGS ROTI maps product towards coverage of the equatorial region and the Southern hemisphere. We present our recent development towards the new ROTI maps product based on the GPS observations from UNAVCO, IGS, CORS, SONEL, and EPN networks.

In order to evaluate extended ROTI maps performance, the ability to represent well-known features of ionospheric irregularity development over the Southern hemisphere and at low latitudes was analyzed. This assessment was performed by estimation and comparisons of patterns of the ionospheric irregularities behavior. For auroral and middle latitudes, we present the interhemispheric cross-analysis of ROTI-based ionospheric irregularities occurrence over the Northern and Southern hemispheres. To demonstrate the performance of the ROTI maps over high/mid-latitudes of the Southern hemisphere, we carried out the comparison study of irregularities development specified by ROTI maps for both the Northern and Southern hemispheres for the case of the recent geomagnetic storm that occurred in February 2022. The diurnal ROTI map for the most disturbed day revealed both the large increase of ROTI magnitude and large-scale spatial expansion of the whole irregularities oval. Such a pattern of the ionospheric response was observed in both hemispheres, but with some interhemispheric differences due to the opposite seasons in the Northern-Southern hemisphere.

For the low latitudes areas, we examined the sensitivity of the resulted ROTI maps to detect plasma irregularities associated with equatorial plasma bubbles development at low, moderate, and high solar activity periods during so-called “bubble seasons”. For all examined periods, the ROTI maps allow to recognize plasma irregularities related to plasma bubble phenomena during local postsunset hours. The most intense and prolonged in time irregularities were developed during the solar maximum period and less pronounced at low solar activity period. The intensity, as well as latitudinal and temporal extensions of EPB-related ionospheric irregularities detected by ROTI maps, are related to the increase of background electron density due to solar flux variations. The ROTI maps product can serve as a useful tool to investigate the ionospheric irregularities occurrence.

Finally, we proposed the new data format for the extended ROTI maps product that based on the actual IGS ROTI maps for the Northern hemisphere and included several additional sections contain gridded ROTI data for the Southern hemisphere and low latitudes region.

## Data Availability

The geophysical parameters data were obtained from NASA/GSFC’s Space Physics Data Facility’s OMNIWeb service (https://omniweb.gsfc.nasa.gov/ow_min.html, accessed on 27 April 2022), Kp index data were available from GFZ Potsdam (ftp://ftp.gfz-potsdam.de/pub/home/obs/kp-ap/tab/, accessed on 27 April 2022) and the AE index data were available from WDC for Geomagnetism, Kyoto (http://wdc.kugi.kyoto-u.ac.jp/ae_realtime/, accessed on 27 April 2022). The DMSP SSUSI results were obtained from the Johns Hopkins University Applied Research Laboratory (http://ssusi.jhuapl.edu/images_daily_l1b, accessed on 27 April 2022). We acknowledge the NASA/GOLD mission science team for providing GOLD data to the public (https://gold.cs.ucf.edu, accessed on 27 April 2022). GNSS data are available with UNAVCO (ftp://data-out.unavco.org, accessed on 27 April 2022), CORS (ftp://geodesy.noaa.gov, accessed on 27 April 2022), IGS (ftp://igs.bkg.bund.de/IGS/obs/, accessed on 27 April 2022), SOPAC (ftp://garner.ucsd.edu), BKGE (ftp://igs.bkg.bund.de/euref/obs, accessed on 27 April 2022).
